# The Physiology, Pathology, and Treatment of Asphyxia

**Published:** 1834-07-01

**Authors:** 


### THE PHYSIOLOGY, PATHOLOGY, AND TREATMENT OF ASPHYXIA. By *James Phillips Kay*, M.D. Octavo, pp. 344. 1834.

THE subject of asphyxia is not quite so hackneyed, in this busy age of authorship, as most other themes of medical literature. It may, therefore, be agreeable, as well as profitable, to our readers, to review some of the leading topics connected with it, as they involve many curious speculations of physiological science, and the deductions which have been drawn from these ought to be well understood by every practitioner. True, indeed, many of us may pass through a long and actively engaged life, without being once called upon to employ the means of resuscitating persons who have been asphyxiated, whether from drowning, hanging, or respiring noxious airs; but such cases may occur, and then, should we be found wanting in proper knowledge and in ready assistance, our characters may be ruined and our inward peace destroyed. Moreover, the same principles which ought to guide us in the treatment of asphyxiated adults, form the basis of all judicious attempts to resuscitate the lives of still-born infants.

Without further preface, we proceed to examine, very briefly, the most approved theories on the subject. It is unnecessary to allude to the speculations which were entertained by physiologists previous to the time of Bichat, that boast and pride of the French school. It was he who exposed the incorrectness of the opinions held by Harvey, Haller, &c. that death from asphyxia was owing to the mechanical obstruction to the flow of blood through the lungs, in consequence of the subsidence and falling together of these organs; and of the hypothesis of Goodwyn, who supposed that the venous blood, when admitted into the left cavities of the heart, acted on their internal surfaces as a sedative poison, and thus caused a paralysis of their motions.

His celebrated experiment of tying the trachea of an animal, and then dividing one of its arterial trunks, satisfactorily demonstrated, that the dark, unaërated fluid continued to permeate freely the heart and general circulatory system—that it was projected per saltum from the opened vessel, and that these phenomena lasted from three to four minutes.

No one has gainsayed the accuracy of Bichat's statements, and we are, therefore, bound to accede to his conclusions. By a series of well-contrived and most ingenious experiments, he was led to propose a theory of asphyxia, which has been almost universally admitted as the true one by physiologists to the present day. Having ascertained that the circulation is carried on for a limited time (from three to five minutes) after the respiratory functions have been arrested, he rightly inferred that every organ and every tissue must necessarily be permeated by the dark venous blood ; and this, he afterwards found, was incapable of supporting the functions and vitality of these organs and tissues. The most important and the most essential to a continuance of life are the brain and heart ; if, therefore, said he, the functions of these can be shewn to be arrested by the transmission of venous blood through their structures, we can have no difficulty in explaining the phenomena of asphyxia. The following experiments were performed :—

" I opened, in an animal, the carotid and jugular ; I received into a syringe, heated to the temperature of the body, the fluid that the latter poured forth, and I injected it into the brain by the former, which I had tied on the side nearest to the heart, to avoid hæmorrhage. Almost immediately, the animal became agitated ; his respiration hurried, he appeared to suffer from dyspnœa similar to that which occurs in Asphyxia : he soon exhibited all its symptoms ; animal life was entirely suspended, the *heart continued still to beat, and the circulation to proceed during half an hour,* at the termination of which, death extinguished also the organic life. The dog was of a middle size, and about six ounces of dark blood were gently injected, lest the effect which resulted from the nature of the fluid should be attributed to a mechanical shock." 190.

And again :—

" Divide in a dog the trachea, and close it so that no air can enter ; at the expiration of two minutes dark blood flows into the system of red blood. If you then open the carotid, and receive into a syringe the blood which issues from the opening, in order to propel it into the brain of another animal, this last soon falls with an embarrassed respiration, sometimes with plaintive cries, and soon dies." 192.

Thus much for the operation of the venous blood on the functions of the nervous system : and so deleterious has this been considered by many physiologists, that they have ascribed the death in asphyxia more directly and immediately to this cause, than even to the paralysis of the heart's actions ; the doctrine which was entertained by Bichat. Beclard, in his Elements of General Anatomy, says—

" Asphyxia, whose cause has been so much sought in the obstruction of the transit of the blood through the lungs—(Haller) in the stagnation of the venous blood in the left ventricle—(Goodwyn) in the penetration of the muscular structures of the heart by this blood—(Bichat) results much rather from the transfusion of the nervous structure by dark blood." 183.

And Dr. Alison, in his Outlines of Physiology, records similar sentiments: —He says :—

" The experiments of Bichat, Brodie, and others have satisfactorily shewn, that a quantity of blood sent from the right side of the heart to the lungs, although not arterialized there, passes on to the left side of the heart, and is propelled into the arteries; and that as soon as this venous blood reaches the brain, the animal becomes insensible, and generally convulsed. It appears from these facts, that in death by Asphyxia, the deleterious influence of venous blood is exerted first on the nervous system ; next, and most fatally, on the circulation in the capillaries of the lungs, and, in a less degree only, on the heart and other muscular parts." 183.

It is by no means so easy to prove satisfactorily by experiments that the venous blood, when admitted into the coronary arteries of the heart, acts so directly, like a poisonous sedative on the contractile functions of the heart, as we have seen that it does on the nervous system. We are obliged to have recourse to analogy; and the analogy has been drawn from observing the effects of the injection of the dark fluid into the arteries of a limb. Bichat assures us, that this operation very generally induces paralysis of the muscles of that limb; and he has therefore inferred that the transmission of dark blood through the coronary arteries of the heart must have a similar sedative effect on the functions of this vital organ:—his words are—

"I am well persuaded that if it were possible to propel by the coronary artery, dark blood, whilst the florid circulates as usual in the aortic auricle and ventricle, the circulation would be almost as soon interrupted as when the blood penetrates the tissue of the heart by the coronary arteries, only after having passed the two cavities of arterial blood." 169.

The leading aim and object of Dr. Kay's work is to prove that many of Bichat's data are not strictly correct, and that his conclusions are inaccurate and fallacious. It would have enhanced the merit of his labours, and rendered his work much more valuable, had a condensed and perspicuous arrangement of his arguments and opinions been diligently pursued. Dr. K. has probably little idea of the irksomeness of the labour which a reviewer, (and in truth every reader ought to be more or less of one in his studies,) has to undergo, in gathering together the important parts of such an essay as his, for the purpose of conveying within a short space the pith and marrow of its contents; else assuredly he would have submitted them over and over again to a mental distillation, till the pure undiluted spirit remained. It is a most crying fault, which almost all authors of the present day are guilty of, that of "hypertrophying" their work. The thoughtful remark of old Selden ought to be ever before them—"Had I had more time, I should have written less."

The objections which Dr. Kay, with very considerable ingenuity, had adduced against the doctrines of the great French physiologist may be aptly divided into those which have reference to the brain and nervous system, and into those which apply to the heart and general muscular system;—and first, respecting the disturbance of the cerebral functions, caused, or supposed to be caused, by the transmission of dark blood through the vessels of the brain, we may adduce the following experiments.

*Exp.* 1. The carotid artery of a rabbit was divided, and a drachm and a half of blood permitted to escape; blood taken directly from the exposed vena cava of another living rabbit was then gently injected towards the brain. This was repeated several times, until about three drachms and a half had been thus introduced. "The animal exhibited no signs of uneasiness whatever, but appeared perfectly well." This might proceed from great care being taken to avoid any great plethora, and to prevent the admission of air along with the blood.

*Exp.* 2. The carotid artery of a rabbit divided, and a drachm and a half of the blood permitted to escape, as in the former experiment. The blood from the vena cava of another rabbit injected. When more than three drachms had been introduced, the animal exhibited no signs of suffering: it remained languid and weak for some hours afterwards, and then recovered.

The other experiments, performed with a similar view, and exactly in the same manner, are admitted by our author himself to be unsatisfactory; (if so, what was the use of recording them?)

And now let us see what are the conclusions which Dr. Kay draws from his enquiries upon this part of the subject.

"They prove (says he) that though venous is a much less nutritious and stimulating fluid than arterial blood, it may circulate through the cerebral mass without producing, by its *contact* with the brain, a sudden suspension of the functions of the nervous system.

I conceive that it must be regarded, as a fluid capable of only slightly nourishing and stimulating the nervous system. Its presence in the vessels of the brain, even for a short time, occasions languor and feebleness, and if its circulation were prolonged, we may imagine that sensation and voluntary motion would become still further impaired; but it does not destroy life by contact with the brain, and in Asphyxia, small quantities of it are transmitted, and for a short period only, to the cerebral structure." 198.

The ready remark which every reader will make is, that all such experiments as we have recorded above are quite unsatisfactory; and why? because the arterial circulation continued free and unobstructed through the other carotid of the animal. We cannot therefore, in canvassing the opinions of Bichat on this part of the subject, permit ourselves to be in any degree influenced by the results of Dr. Kay's experiments; and if we proceed further in this enquiry, and examine the objections which the latter has proposed to the reasoning of the former, we shall find that the dispute is rather about the meaning of a word than upon matters of fact, or the accuracy of the conclusions to be deduced from them. For this purpose, we shall relate an experiment recorded by Bichat, and append Dr. Key's comments on it.

"Whilst Asphyxia is in its first stage in an animal, I have observed that by transfusing towards the brain red blood, by means of a tube adapted to the carotid of another animal and to its own, motion gradually revives, the cerebral functions are in some degree re-excited, and even often sudden agitations of the head, the eyes, &c., announce the first contact of the blood; but these good symptoms *soon disappear, and the animal relapses, if the cause of the Asphyxia continue, if, for example, the stop-cock fixed in the trachea remained closed.*" 202.

Dr. K. observes—

"The supply of blood from the heart to the brain being, as we have shewn, speedily interrupted in Asphyxia, nothing can so immediately revive the sensorial functions of the animal, as the transfusion of arterial blood from the carotid artery of another animal towards the cerebral mass. But if dark blood *extinguished* the functions of the brain by its poisonous qualities, we cannot conceive that its expulsion from the tissue could be so rapidly completed as to permit the sudden re-animation which occurs in the experiment.

If however we conceive dark blood to be only a fluid *exceedingly less capable of nourishing and stimulating the nervous system* than arterial blood, we may discover how, when it has circulated through the brain, the propulsion of a more nutritious and stimulating fluid into the tissue, may revive the phenomena of life." 202.

From this it appears that the sum and substance of Dr. Kay's objections is, that "the dark blood does not *extinguish* the functions of the brain by its *poisonous* qualities," but "is only less capable of stimulating the nervous system." Bichat considered it as a direct sedative; Dr. K. views it as a minor stimulant.

It seems to us quite unnecessary to have devoted so much space and time to the settling of so small a dispute; although we admit that Dr. K. is more correct in his phraseology. His sentiments are well expressed in the following extract:—

"When respiration is suddenly arrested, the first symptoms of disturbance of the cerebral faculties ensue as soon as the quantity of fluid circulating is decidedly diminished, and motion and sensation are at an end before the circulation ceases. These effects we conceive to be owing to two circumstances, first, the *quantity* of blood circulating in the arteries is exceedingly *diminished,* and secondly, that fluid has lost, in a great degree, its arterial character, and has therefore become *less capable of maintaining the perfect phenomena of life, and especially the organic motions of the nervous system.*" 203.

And in support of that part of his doctrine which is expressed by the words "the quantity of blood circulating in the arteries is exceedingly diminished," he alludes to the symptoms observed in excessive hæmorrhage, in which, as in asphyxia, the supply of blood to the brain is gradually diminished;—these are, to use the words of Dr. Blundell, "dyspnœa, struggling, cessation of the circulation, relaxation of the abdominal muscles, and a complete asphyxia." The series of masterly experiments performed by Edwards (a very extended review of whose admirable work appeared in a late No. of our Review) demonstrates most unequivocally that venous or asphyxiated blood, "though incapable of supporting the life of reptiles and of young mammiferous animals, for an indefinite period, exerts no directly or immediately poisonous influence on the nervous system, so as to destroy its powers by mere contact, like a foreign fluid." Had we no other argument, we are of opinion that the one afforded by these experiments is quite conclusive against Bichat's theory in its full extent; and must force us to agree with Dr. Kay, that—

"In asphyxia, the imperfectly arterialized blood which circulates through the brain, is less conducive to the maintenance of its functions than arterial blood: but those functions and life are abolished, because the circulation is arrested in the lungs when the respiratory phenomena cease. The fluid which is then propelled into the nervous tissue, though less capable of developing its powers than arterial blood, can maintain for a time imperfect motions of the vital parts, and contribute to the evolution of an inferior order of vital phenomena." 207.

We now proceed to the investigation of the effects produced by venous or asphyxiated blood, on the functions of the muscular system generally, and of the heart in particular. This enquiry is one of great interest; and its accurate determination must be accomplished before we can admit the truth of the theory of asphyxia, which ever since the time of its proposer, has been almost universally assented to. We have already seen that Bichat has asserted "that venous blood injected into the main artery of a limb caused its muscles to become very sensibly feebler, and sometimes even immediately paralytic." He states, also, that the ligature of the artery, especially if that be the aorta, paralyses the limbs; but he does not attempt to shew, that the paralysis occurs earlier when dark blood is injected than when the main artery is simply tied. The loss of mobility may therefore possibly be owing rather to the interrupted transmission of the arterial blood, than to any sedative operation of the venous. Dr. Kay performed numerous experiments to ascertain this point.

For example, he tied the aorta and one of the common iliacs of a rabbit, and injected blood from the vena cava of another rabbit into the other iliac; the injection was thrice repeated in the course of 30 minutes. No difference could be discovered in the contractility of the muscles in the two extremities: the contractility was ascertained by inserting needles attached to the wires of a galvanic battery.

So much for the effect of the venous blood of another animal. Dr. Kay then experimented with the blood of an animal which had been asphyxiated by tying its trachea, and he obtained the same results as in the former experiment: viz. that the muscles of that limb which was so treated, retained their contractility, as long as those of the other in which the circulation was arrested. He varied the experiment thus:—the trachea of one rabbit was tied; and in another, the chest was opened and the heart tied at its base. The muscles continued contractile an equal time in both.

These experiments are therefore quite at variance with the statements made by Bichat; and additional proofs of their incorrectness may be derived from the following circumstance related by our author:—

"It is remarkable," says he, "that though the presence of venous blood in the tissue of muscle has been considered by Bichat and others to be fatal to its contractility, when the vein only is tied, or the artery and vein together, preventing the egress of this blood, the phenomenon does not so soon cease as in the simple ligature of the artery." 141.

From these data, Dr. Kay draws the conclusions, that the supply of dark blood from the left heart in asphyxia has no positive and direct noxious influence on the muscles; that they are affected in exactly the same degree as when the supply of blood to their tissue is interrupted; [if there be any difference in the two cases the contractility remains for a longer time in the former than in the latter case] and that therefore the analogy which Bichat has drawn from the influence of venous blood upon the voluntary muscles is without foundation, and cannot be applied to explain the cessation of the heart's movements.

The next step in the investigation is to endeavour to ascertain the effects of venous and of asphyxiated blood on the heart itself. This organ, it is admitted by all, retains its contractility for some time after that of the muscles has ceased. The right auricle and ventricle in common death always continue to contract longer than their corresponding systemic cavities, and the venous sinus and auricle longer than the ventricle; the right auricle is therefore the "ultimum moriens." The philosopher is naturally anxious to find out the cause of these differences; why the one side of the heart should retain its contractility longer than the other, and why the right auricle should survive the left one. As far as we can determine by anatomical examination their texture is the same; their bloodvessels and nerves come from the same source, and the only difference which we can perceive between them after death is, that the right is almost always distended with blood, and the other is empty, or nearly so. Haller supposed that, as the blood is the natural stimulus of the heart, the cavities which become empty first, must first lose their power of movement; and he has related the following experiment in confirmation of his ideas.

"I divided the venæ cavæ, and emptied the right auricle and ventricle, afterwards tying the veins. I opened also the pulmonary artery by an ample wound, that the right ventricle might more easily discharge its blood, so that the right auricle and ventricle evacuated their contents; on the other hand I left the pulmonary veins free, but placed a ligature on the aorta, so that the left auricle and ventricle received blood, but could not expel it. The left auricle and ventricle always continued acting longer than their corresponding cavities, even to the fourth hour. The right auricle was in perpetual quiescence. The right ventricle contracted either imperfectly, languidly, or a shorter time, when it was not altogether immotile; or its quiescence was perfect, as often as the experiment had so succeeded, that the cavities were entirely evacuated." 157.

It will be perceived that Goodwyn's theory of asphyxia accords well, and receives much support from this experiment of Haller; and it was doubtless the consideration of such facts that gave origin to it; and very naturally and perhaps correctly so. Indeed it has long been our decided conviction, that on this, as upon a number of other physiological enquiries, much error has frequently been committed by an over-fond and inconsiderate zeal of discovering a single, and as it were, an unassisted cause of the phenomena of living matter. Surely we should be led, a priori, to take a very different view;—an animal and a plant have more than one organ and one system in their wonderful economies, and we know that all the complex machinery of their functions is intimately and indissolubly woven together to form one admirable whole. Is it therefore at all improbable that the different parts of the sanguiferous system are differently affected by the two kinds of blood circulating through the body? that the one from being as it were accustomed, is impressionable only by the stimulus of the venous, and the other by that of the arterial blood; and therefore that the left side of the heart very possibly may be incommoded by the presence of dark blood within its cavities even although the coronary arteries still transmitted scarlet blood to its tissue, and the nervous system retained at the same time all its normal energies. This indeed is mere conjecture, and can never be proved by direct observation or experiment; but the same objection may be made to Bichat's theory, and also to the one, which we shall immediately find is proposed by Dr. Kay; and it is here especially worthy of notice that Dr. Kay's own theory is built on the very same fundamental principles as Goodwyn's, viz. the incapacity of certain parts of the circulatory apparatus being stimulated by dark-coloured blood. We shall only mention at present one sentence.

"In asphyxia, the blood, from the want of a supply of air to the lungs, rapidly loses its arterial character, until at length it becomes so much deteriorated, that it is incapable of exciting the organic sensibilities of the pulmonary vessels, which therefore refuse to transmit it through the tissue." 236.

Like all his predecessors our author displays the utmost ingenuity in trying to make every fact and phenomenon subservient to and accordant with his own doctrines; and he seems to forget that they may often be quite as consistently explained on the theory of others. For example, the manner in which he accounts for the transference of a more abiding contractility, from the right to the left cavities of the heart, is by supposing that the exit of the blood contained in the coronary veins is prevented when the cavities are full, and occurs only when they are empty; that therefore the veins of one side remain gorged, and of the other side are drained; and that this very engorgement or congestion is in some degree favourable to the persistence of the contractility of the muscular fibres. In ordinary cases the right cavities are full and the left are empty; hence, according to Dr. Kay, the coronary veins of the former retain their blood; and those of the latter are deprived of it. When however an experiment like that of Haller's is performed, circumstances are reversed, and the left side cavities remain longer contractile than the right. The cessation of the heart's contractions, according to him, seems, therefore, to be owing quite as much to the want of a proper supply of blood to its texture, as to any noxious influence of the venous or asphyxiated fluid.

Our author proceeds to illustrate the subject, by a very extended reference to the experiments of Edwards, which we have previously alluded to. The results of these are too important to be passed over; and, as they tend to throw considerable light on the hitherto obscure theme of asphyxia, we shall briefly detail a few of the most important.

1. Reptiles, in which the circulation has been destroyed by excision of the heart, continue to live much longer when exposed to the air than when placed in water. A frog, whose heart was cut out, was immersed in water; when life seemed to be extinct, it was taken out and exposed to the air—it revived, and lived for some time. We are hence led to infer the pernicious effects of plunging asphyxiated animals in warm baths, for the purpose of recovering them; the contact of the vivifying air to the skin being thus effectually prevented.

2. Frogs, which were asphyxiated by pieces of bladder tied tightly over their heads, lived in air from one to five days, whereas, in water, they died in the course of ten or twelve hours. This experiment is confirmatory of the conclusion drawn from the preceding one, and very satisfactorily illustrates the enlivening influence of the air on the blood, through the medium of the skin.

3. The young of many mammiferous animals can bear, without loss of life, the deprivation of air for a considerably longer period than the adults. Buffon was the first to announce this curious difference; and almost every person knows his ingenious experiment of making a bitch litter in water: the pups, on being born, were immediately transferred from the water to a tub of warm milk, and left there for half an hour; then permitted to breathe for half an hour, and again immersed for the same period; and yet they lived, and were as healthy as other puppies produced by the same bitch, after she had been removed from the water. Le Gallois obtained similar results with rabbits, and he ascertained that the power of enduring the want of air rapidly diminishes after birth: new-born rabbits lived for 28 minutes—when five days old, for 16 minutes, and when fifteen days old, they did not live longer than the adult animals.

4. The duration of life in young animals, when deprived of air, is much influenced by the temperature of the surrounding medium. Young kittens, plunged into water at 32° F. died in four minutes and a half—at 57° F. in ten minutes and a half—at 68° F. in thirty-eight minutes—at 86° F. in twenty-nine minutes—at 104° F. in ten minutes and a half.

Hence it appears, that the two extremes of temperature are equally unfavourable, and we thus perceive the danger of rashly applying a high heat to restore asphyxiated persons.

The consideration of the preceding statements leads us to this important conclusion, that in the inferior classes, and in the young of warm-blooded animals, the circulation of venous blood is not positively and directly noxious, as Bichat imagined, but is capable of supporting life for a considerable period after respiration has been suspended. It may be said, and said justly, by the disciples of Bichat, that the argument which we have just adduced rests upon an unsatisfactory analogy—that the constitution of the young animal may differ very materially, in many of its capabilities, from those of the adult, and that reasoning drawn from the one is not strictly applicable to the other. We admit, to a certain extent, the justness of these objections; still it cannot be denied that the analogies, such as they are, disfavour Bichat's theory. A phenomenon observed by Dr. Kay, in several of his experiments, may also be adduced here. He found that the heart continued to act vigorously in an asphyxiated animal for a minute or two after the blood had ceased to flow from the divided aorta. We cannot well reconcile this occurrence with Bichat's doctrine; and we may, therefore, accord to our author the merit of having proved that, "though the venous blood is less capable of maintaining the vitality of the body than the arterial blood, it exerts no positively noxious, and, as it were, poisonous, influence on its functions."

Our readers may now ask, what theory has Dr. Kay substituted in the stead of Bichat's. We shall give it in his own words:—

"As this change (the gradual conversion of the bright vermilion blood into the dark-coloured venous, when respiration is obstructed) proceeds, the circulation becomes more feeble and slower at each successive step of the change, until when venous blood enters those vessels which formerly conveyed arterial blood only, this degenerated fluid is no longer able to excite their action, and the circulation stagnates in the structure of the lungs. The pulmonary veins then discharge their last meagre supply through the left auricle into the left ventricle, which propels its last and feeblest tide into the arteries, in which the circulation has, every moment, become more scanty, until the pulsation has gradually been extinguished." 21.

Again:—

"The obstacle to the circulation does not therefore exist in the heart, but in the lungs." 22.

And, lastly, as—

"The general conclusions concerning Asphyxia obtained from this investigation are, first, that the circulation is arrested after respiration ceases, because, from the exclusion of oxygen, and the consequent non-arterialization of the blood, the minute pulmonary vessels which usually convey arterial, are then incapable of conveying venous blood, which therefore stagnates in the lungs. Secondly, that the arrest of the circulation is sudden when the lungs are entirely deprived of air, and that blood ceases to flow from them into the left cavities of the heart, even in the smallest quantities, in about three minutes and a half. Thirdly, that even supposing a great quantity of venous blood were transmitted through the lungs, and permeated the heart and muscles, it would not impair their contractility: but on the contrary, that it is even capable of supporting this power for a certain period. Venous blood does not possess any noxious quality, by which the organic functions of these tissues can be destroyed, but is simply a less nutritious and stimulating fluid than arterial blood. The functions of muscular fibre cease in Asphyxia, because the circulation, and consequently that supply of vital fluid which is necessary to life, is arrested in the lungs." 182.

Dr. Kay has been more fortunate in exposing the insufficiency of the doctrines of others than in establishing the truth of his own; and so we predict it will always be, when physiologists endeavour to find out one, and *only* one, cause for any of the phenomena of living matter. There is not a single argument adduced to prove, that "the innervation or organic sensibilities of the minute pulmonary vessels" are sooner affected by, or are more hostilely opposed to the transmission of the dark-coloured blood than those of the capillaries in every other part of the system :—They are all, we have reason to believe, equally so, both in point of time and of degree ; and we should say that, in death from asphyxia, the mortal changes are not nearly so consecutive to each other as physiologists have taught us, but rather that they are simultaneous and co-operating ; and that, with but a very short interval, the brain and nervous system are paralyzed, the heart and its thousand pipes are robbed of their moving powers, and the commencement of death has taken place in every part.

We now dismiss the subject of the physiology of asphyxia; and, before adverting to the means of resuscitation, we shall briefly allude to a topic of much interest—one, the consideration of which is very intimately blended with that of asphyxia—we allude to the phenomena of syncope. It is no very easy task to point out accurately all the differences between these two states of suspended animation. This fact we know, that the one is much more speedily fatal than the other; and hence we have, *à priôri*, strong reason to infer that their pathological conditions are different.

It may be stated as a general law, subject to very few exceptions, that the interruption of the respiratory function proves fatal to adult mammiferous animals in from three to five minutes. Such we observe to be about the limits, in animals which are subjected to experiment, and such, we have reason to believe, has been the result of enquiries on the subject of accidental drowning. The period for which life may continue, during a state of syncope, cannot be well discovered; but we may certainly say, at the least, for several hours. This very striking contrast is not controverted or nullified by the histories of those remarkable cases, in which persons, we are told, have been recovered after they had been under water for upwards of one, and even of many hours. True it is, that not a few of these histories must be regarded as fabulous, and are found in those collections of marvels and wonders which were in vogue some 80 or 90 years ago. But, admitting the authenticity and correctness of some of these cases (and the characters of the vouchers are too respectable to allow our doubts to the contrary), it has been most reasonably supposed that they were, in truth, not examples of genuine asphyxia, but of asphyxia conjoined with a state of syncope.

The terror and agitation, at the moment, may very probably so overwhelm the poor sufferer, that he sinks into a state of complete syncope ere yet he has met his danger. The effect of this must be, to render him quite insensible to suffering, and to prevent those writhings and dreadful struggles which attend asphyxia under other circumstances, and which necessarily have a great influence in causing that excessive accumulation of blood in the large veins and the right side of the heart, which is invariably found on dissection. In syncope, on the other hand, there is no such congestion in one part of the system and vacuity in the other; there is rather a sudden arrest, or a very feeble and imperfect movement of the sanguineous fluid; probably in the same degree at every part: and besides this, and perhaps as a consequence of it, the blood does not undergo the same changes so rapidly, and become so soon unfit for the purposes of life, as when the breathing is suddenly stopped. In syncope, the brain and nervous system are first and primarily affected; at least this is the case in by far the greater number of instances—they are stricken, as it were, momentarily dead, and the animal has not one of its animal functions remaining; all is gone at once, save the mere organic or vegetable life, and this only in an imperfect degree remains; but as long as that spark exists, we may expect to be able to resuscitate the whole; for the machinery of the system, though it has almost ceased to move, is entire, and only requires one sudden impulse to set it again going: but not so in asphyxia;—besides the arrest of the functions, there is a positive mischief inflicted on every part of the system; the one-half of the circulatory apparatus is empty, the other half is gorged, the functions of the brain and the rest of the nervous system are extinguished, or nearly so; in part from a deteriorated blood contained in some of their vessels, and in part also from an actual diminution in the quantity of it in others; and so are those of all the glands and other viscera of the body; here, then, we see that the wheels of the machine have received a positive injury, far beyond that which occurs in the other case. Hence it is, that attempts at resuscitation are so much less successful in the one than in the other of these cases. The following remarks of our author deserve insertion.

"In animals, life can seldom or never be restored when five minutes have elapsed after respiration has been arrested, and it is therefore most probable, that the remarkable resuscitations, reported to have taken place after a much longer duration of apparent Asphyxia, have occurred in forms of syncope having few if any of the characteristics of Asphyxia. Leroy considers it desirable that some means of distinguishing syncope from Asphyxia should be discovered. It is most probable, however, that, when syncope has continued a considerable period, the state of the capillary circulation may acquire more of the characteristics of Asphyxia, and that some congestion may occur in the large veins. In the absence of any correct means of diagnosis, it is proper to neglect all attempts to distinguish the two diseases, and to employ for the restoration of all cases of suspended animation, with caution but with zeal, the means used in Asphyxia." 239.

Allied in some respects to that form of asphyxia which we may designate the "A. Syncopica," is that form of it which occurs in children at the birth from a variety of circumstances, as compression of the cord, premature separation, entire or partial, of the placenta, &c. Except in cases of violent flooding, there is in general no diminution in the quantity of the blood, but this is no longer supplied with that vivifying something which it receives from the placenta, just in the same manner as the venous blood of the adult is enlivened by exposure to the air in the lungs; the interruption, therefore, of the communication in the one case, and of the admission of air in the other, places the beings in very similar conditions, at least as far as the physiology of these conditions is concerned—not, certainly, in their capabilities of being resuscitated. The difference in this latter respect we alluded to, when mentioning the results of Edwards' experiments. Gardien disputes the propriety of applying the term asphyxia to the still-born condition of infants, and denominates it syncope, because it commences, in his opinion, with the heart, which is deprived of its accustomed supply of blood; but he is decidedly in error, when he considers pulselessness as the chief sign of this state. Burns, Desormeaux, and the majority of obstetrical authorities, most distinctly assert, that the umbilical chord, in these cases, sometimes continues to pulsate for a short time before respiration has commenced. Whether the idea of M. Fretau, that pressure on the cord may interrupt the flow of blood in the umbilical vein, without obstruction to that in the arteries, is ever correct or not, it is almost impossible to say; at least we have no data to warrant our assent.

In cases of flooding, before the birth of the child, there can be little doubt that its system may be most seriously drained of its circulating fluid, and a state of genuine syncope may be thus induced. Under such circumstances, the death of the infant is to be explained in exactly the same manner as we should explain death from syncope in the adult; but the etiology of asphyxia, at the two different periods of life, is probably modified by the differences existing in their respiratory and circulatory apparatuses, and, we should perhaps add, of their nervous systems. Our reason for including the nervous system is, because the generation of animal heat is very generally admitted by physiologists to be most intimately dependent upon a healthy state of its functions; and we are taught by Edwards, that the capability in animals of retaining life, when the respiration is prevented, is, on all occasions, inversely proportionate to their power of supporting their normal temperature. In the infant, this power is inferior to that in the adult; and hence it may be longer asphyxiated with impunity than the latter.

Our remarks on the treatment of asphyxia will be very short, and we shall confine them to what may be deemed the novelties of the subject. One of the practical deductions which Dr. Edwards has drawn from his experiments (so often alluded to), is the importance of exposing the body of an asphyxiated person freely to the air, whose temperature ought to be always of a moderate elevation. The practice of clothing the body, or of immersing it in a warm-bath, is decidedly pernicious, as the skin is thereby excluded from the vivifying influence of the atmosphere; and the application of blankets wrung out of hot water, of hot bricks, &c. may have the effect, indeed, of more powerfully exciting the nerves of a part; but this effect is only temporary, and gives way to a corresponding exhaustion of their energies. The best mode of applying heat is in the following method, directed by the Royal Humane Society.

"The natural and kindly warmth of a healthy person, lying by the side of the body, has been found, in some cases of adults, and particularly of children, very efficacious." 39.

A topic of still higher importance to be well understood by every medical man, is the proper method of insufflating the lungs. It is unnecessary, we hope, to condemn the practice of opening the trachea, for the purpose of introducing any tube, except under very peculiar circumstances—no one ought to pretend to the name of a surgeon who cannot insert it through the glottis. M. Leroy has contrived an instrument to facilitate this operation; but the simple manœuvre of pulling forwards and depressing the tongue will open the glottis sufficiently; and if we make pressure on the pomum Adami while we pass the instrument along, it will be prevented from entering the

œsophagus. In inflating the lungs, especially with the bellows, great caution is requisite that the air is not forced in with undue violence, else the pulmonary cells may be ruptured, and fatal consequences ensue.

"Leroy discovered that brisk inflation of air into the trachea killed rabbits, foxes, goats, sheep, and other animals, even when the force employed was that of an expiration from the human lungs." 226.

And we have the following melancholy announcements, on the authority of Majendie, that—

"From the records kept in the city of Paris of the results of means employed for the recovery of persons drowned, the greater prevalence of the practice of insufflation has been coincident with a decrease of the number restored to life." 224.

And that—

"The most limited conclusion to be deduced from these facts is, that insufflation of the lungs, as it has been recently performed in attempting to restore cases of Asphyxia, has not increased the efficiency of the means employed for this purpose, and may even have diminished the probability of resuscitation." 225.

M. M. Leroy and Majendie found, on the examination of those animals which had been killed by the brisk insufflation of air into the trachea, air in the cavities of the pleura, and sometimes bubbles of air throughout the whole sanguiferous system; and as their experiments were repeated on human corpses, with the same results, we at once perceive the dangers of incautious attempts to resuscitate asphyxiated persons. With the view of regulating the quantity of air admitted into the lungs at each inflation of the bellows, M. Leroy recommends that their handles should be provided with a graduated scale, by which the quantity can be accurately determined. The simultaneous raising and depressing of the chest of the patient by means of his bandage, may powerfully contribute to the restoration of breathing. As this apparatus is not very generally known to the profession, we shall extract Dr. Kay's description.

"A piece of strong flannel, an old blanket, sheet, or other cloth—most easily to be obtained at the moment—is to be cut of the following size, and in the following manner. It should be six feet in length, and in breadth eighteen inches. Six strips are then to be cut or torn lengthwise on each side. Each strip is to be three inches broad, and two feet long. The untorn portion (two feet in length and eighteen inches broad) is to be placed under the back of the patient, from the arm-pits to the upper part of the thigh bones. The strips are then to be brought together over the chest and belly, interlacing each other from the opposite sides, as the fingers are interlaced in clasping the hands. The strips thus arranged are to be gathered into a bundle on each side, and if they are then drawn in opposite directions by two assistants, the edges of the bandage will be made to approach, and firm and equal pressure be produced on the chest and belly of the patient.

The assistants should thus compress the body of the patient by drawing the bandage in opposite directions, and should then relax it, permitting the chest to re-expand, and performing this process at the rate of twenty-five times in the minute." 57.

In concluding our notice of Dr. Kay's work, it would have been gratifying to us had we been able, conscientiously, to approve of it *in toto*. There are, however, many glaring blemishes, which might easily have been avoided by careful and repeated revision; the prolixity and iteration of details are sometimes almost quite unbearable, and this annoyance is enhanced by the neglect of all precision in the arrangement, and by the introduction of much superfluous matter. Perhaps these errors might have arisen from the author having, at some period, intended to deliver the contents in the form of lectures to an audience :—Are we right in the conjecture ? A diligent, and, we may add, a laborious examination of the work, has prompted the suspicion.

That the arguments which Dr. Kay has adduced, satisfactorily demonstrate that the theory of Bichat, in its full extent, is not correct, and that the proximate cause of the arrested circulation in asphyxia is to be sought for, rather in the obstruction to the pulmonary current than in the paralysis of the heart's action, we willingly and cheerfully admit: but it is necessary to state that he has been anticipated in most of his conclusions, and in the general scope of his reasoning, by Dr. David Williams, of Liverpool, who published, in the Edinburgh Medical and Surgical Journal for Oct. 1823, an ingenious and well-written paper, " On the Cause and the Effects of an Obstruction of the Blood in the Lungs." We have purposely omitted to allude to Dr. W.'s claims before, unwilling, as we always are, to mix up the discussions of personal controversy with the exposition of scientific details.

The following corollaries, announced in the paper referred to, sufficiently substantiate the priority of the author's title.

" 1*st*, The blood is obstructed in its passage through the lungs, on suspension of respiration, while its circulation through the other parts of the body continues.

2*nd*, The obstruction of the blood in the lungs, on suspension of respiration, is not the effect of a mechanical cause.

3*rd*, The obstruction of the blood in the lungs, on suspension of respiration, arises from a deprivation of pure atmospherical air.

4*th*, The blood which is found *post mortem* in the left auricle and ventricle, is the remnant after the last systole, and the subsequent draining of the pulmonary veins.

5*th*, The obstruction of the blood in the lungs, on suspension of respiration, is one of the principal causes of the vacuity of the system circulating arterial blood *post mortem*.

6*th*, The immediate cause of the cessation of the action of the heart, is a privation of its natural stimulus, arising from the obstruction of the blood in the lungs." 530.

Our limits preclude any further remarks; but the preceding extract must be considered altogether conclusive. The illustration of each corollary is given at length in Dr. W.'s memoir, which we have much pleasure in recommending to the attention of all future enquirers on the subject of asphyxia. Unfortunately it seems to have escaped the notice of Drs. Roget and Copland, as no reference is made to it, either in the Cyclopædia, or in the Dictionary of Practical Medicine, under the article Asphyxia.

